# Metadata harmonization–Standards are the key for a better usage of omics data for integrative microbiome analysis

**DOI:** 10.1186/s40793-022-00425-1

**Published:** 2022-06-24

**Authors:** Tomislav Cernava, Daria Rybakova, François Buscot, Thomas Clavel, Alice Carolyn McHardy, Fernando Meyer, Folker Meyer, Jörg Overmann, Bärbel Stecher, Angela Sessitsch, Michael Schloter, Gabriele Berg, The MicrobiomeSupport Team, Paulo Arruda, Thomas Bartzanas, Tanja Kostic, Paula Iara Brennan, Bárbara Bort Biazotti, Marie-Christine Champomier-Verges, Trevor Charles, Mairead Coakley, Paul Cotter, Don Cowan, Kathleen D’Hondt, Ilario Ferrocino, Kristina Foterek, Gema Herrero-Corral, Carly Huitema, Janet Jansson, Shuang-Jiang Liu, Paula Malloy, Emmanuelle Maguin, Lidia Markiewicz, Ryan Mcclure, Andreas Moser, Jolien Roovers, Matthew Ryan, Inga Sarand, Bettina Schelkle, Annelein Meisner, Ulrich Schurr, Joseph Selvin, Effie Tsakalidou, Martin Wagner, Steve Wakelin, Wiesław Wiczkowski, Hanna Winkler, Juanjuan Xiao, Christine J. Bunthof, Rafael Soares Correa de Souza, Yolanda Sanz, Lene Lange, Hauke Smidt

**Affiliations:** 1 Institute of Environmental Biotechnology, Graz University of Technology, Graz, Austria; 2 2Soil Ecology Department, Helmholtz Centre for Environmental Research (UFZ), Halle (Saale), Germany; 3 3German Centre for Integrative Biodiversity Research (iDiv) Halle–Jena–Leipzig, Leipzig, Germany; 4 Functional Microbiome Research Group, Institute of Medical Microbiology, RWTH University Hospital, Aachen, Germany; 5 Helmholtz Zentrum München, Oberschleissheim, Germany; 6 Computational Biology of Infection Research, Helmholtz Centre for Infection Research, Braunschweig, Germany; 7 Braunschweig Integrated Centre of Systems Biology (BRICS), Technische Universität Braunschweig, Braunschweig, Germany; 8 German Center for Infection Research (DZIF), Hannover-Braunschweig site, Hannover, Germany; 9 Cluster of Excellence RESIST (EXC2155), Hannover Medical School, Hannover, Germany; 10 Universität Duisburg-Essen, Essen, Germany; 11 Leibniz Institute DSMZ German Collection of Microorganisms and Cell Cultures, Braunschweig, Germany; 12 Technical University of Braunschweig, Braunschweig, Germany; 13 Max Von Pettenkofer Institute of Hygiene and Medical Microbiology, Faculty of Medicine, LMU Munich, Munich, Germany; 14 German Center for Infection Research (DZIF), Munich, Germany; 15 Bioresources Unit, AIT Austrian Institute of Technology, Tulln, Austria; 16 Leibniz-Institute for Agricultural Engineering Potsdam (ATB), Potsdam, Germany; 17 University of Potsdam, Potsdam, Germany

**Keywords:** Microbiome, Omics technologies, Metadata, FAIR, Repositories, Data storage, Convention on biological diversity

## Abstract

**Background:**

Tremendous amounts of data generated from microbiome research studies during the last decades require not only standards for sampling and preparation of omics data but also clear concepts of how the metadata is prepared to ensure re-use for integrative and interdisciplinary microbiome analysis.

**Results:**

In this Commentary, we present our views on the key issues related to the current system for metadata submission in omics research, and propose the development of a global metadata system. Such a system should be easy to use, clearly structured in a hierarchical way, and should be compatible with all existing microbiome data repositories, following common standards for minimal required information and common ontology. Although minimum metadata requirements are essential for microbiome datasets, the immense technological progress requires a flexible system, which will have to be constantly improved and re-thought. While FAIR principles (Findable, Accessible, Interoperable, and Reusable) are already considered, international legal issues on genetic resource and sequence sharing provided by the Convention on Biological Diversity need more awareness and engagement of the scientific community.

**Conclusions:**

The suggested approach for metadata entries would strongly improve retrieving and re-using data as demonstrated in several representative use cases. These integrative analyses, in turn, would further advance the potential of microbiome research for novel scientific discoveries and the development of microbiome-derived products.

**Supplementary Information:**

The online version contains supplementary material available at 10.1186/s40793-022-00425-1.

## Introduction

The new age of modern “omics” technologies resulted in a tremendous amount of data generated by an ever-increasing number of high-throughput processing methods for microbiome research (Schneider and Orchard 2011; [36]. Omics technologies are defined as high-throughput biochemical assays that measure comprehensively and simultaneously molecules of the same type from a biological sample. They can be based on metagenome, metatranscriptome, metaproteome, or metabolome approaches [5]. Metabolomics experiments generally result in more complex and less defined datasets, therefore we focus here on standards and concepts for DNA, RNA and protein sequence data.

To date, thousands of datasets derived from microbiome research have been stored in diverse public and private repositories. Recent estimates indicate that the global market for microbiome sequencing will continue growing in the next years [35]. Despite this impressive amount of data, the outcome in terms of products from microbiome research both in environmental sciences as well as in the medical field is still limited. In January 2022, only 8000 microbiome-related patents have been registered worldwide (https://worldwide.espacenet.com/patent/). This low number is due to the fact, that most datasets are used only by the researchers that have generated the data for their particular study, and, due to the relatively young field of research [5]. Thus, despite the availability of data in public depositories, microbiome research is scattered into showcases, and a generalization of findings by performing integrative analyses is difficult.

Although comparisons of different datasets are still rare, tools based on artificial intelligence approaches and especially machine learning, which can be applied, develop fast. This would allow nowadays assessments of large datasets and predictions for microbiome assembly as shown for the soil and gut microbiome [3, 11]. In addition, comparative analyses of datasets obtained by different technologies are rare although especially combinations of different methods can increase the accuracy of results in microbiome research [5]. Method development and corresponding standardization and vice versa is a continuously ongoing process. The main reason for this situation is a lack of common standards for data and metadata in microbiome research which is further exacerbated by missing or insufficient metadata in general [2].

While substantial efforts were made to introduce standards for “wetlab work” in microbiome research in the last decades [10], including protocols for DNA extraction (ISO 11063 [37, 45], storage of samples, primer use [40] and bioinformatics [42, 43], so far efforts to define minimum requirements for metadata in microbiome research are comparably rare. Metadata are “data about data” [17]. Generally, metadata should include experimental or monitoring details as well as technical and analytical methods used. In the ideal case, metadata should provide all necessary information to repeat a study or to resample at later stages, on the one hand and should enable researchers to reuse data in a broader context going beyond individual studies, on the other hand. In interdisciplinary, large research consortia such as biodiversity exploratories or medical cohorts, the collection of metadata is often better organized than in single research groups [58],Pinilla-Redondo et al. 2021). One of the main reasons for the lack of commonly agreed metadata minimum requirements is the fact that various groups working with microbiome-related data, such as scientists from basic research and industry sectors, medical doctors, etc., have different demands for their metadata. In addition, they might have to deal with various legal issues that can include data protection or intellectual property right issues.

This publication summarizes the discussions of a series of webinars organized by Graz University of Technology (Austria) and Helmholtz Zentrum München (Germany) in May 2020 on the topic of the importance of metadata in microbiome research. The webinar was co-hosted by the European project MicrobiomeSupport (www.microbiomesupport.eu) and the Initiative for the Critical Assessment of Metagenome Interpretation (CAMI, Germany; [24, 48] and initiated an ongoing discussion on the topic. The online workshop titled “Elaboration of standards and making use of existing data” brought together over 70 researchers and industry partners from all over the world with expertise in plant pathology, soil science, microbe-host interactions, computational microbiology, and microbial ecology. The participants of the webinar were experts in different medical, host-associated, food- and environmental microbiome research areas.

## Main text

Here we discuss the most important questions connected with metadata, which were identified as the following: do we need minimum requirements or standards; which ethical and legal issues of the metadata have to be considered; do we need a common digital identifier and ontology, and how can we implement the FAIR concept, which includes the four principles “findable”, “accessible”, “interoperable” and “reusable” of data [56], while considering legal issues. In an additional chapter, we present selected use cases demonstrating added value of re-using microbiome datasets.

## Minimum requirements or standards?

According to ISO, a metadata standard is “a high level document which establishes a common way of structuring and understanding data, and includes principles and implementation issues for utilizing the standard” (Organisation Internationale de Normalisation, 2013). However, in reality “standards” for metadata requirements differ depending on the group of scientists who propose the “standards”. For example, metadata associated with human pathogens has been standardized by the “National Institute of Allergy and Infectious Diseases”, the “Genome Sequencing Center” and the “Bioinformatics Resource Center Project [12]. Metadata standards have also been recently suggested for agricultural microbiome research [13], for microbiomes associated to food safety (Griffiths et al. 2017) as well as human health (www.stormsmicrobiome.org). When comparing these different concepts for metadata, we found that only the metadata fields that were common among all selected repositories were the project name and the date of sample collection.

Therefore, the definition of a unifying metadata standard is contrasting the different metadata standards, which have been proposed for various disciplines in microbiome science. This challenges the question whether a metadata standard is needed or whether minimum requirements are the better option to fulfill the demands of microbiome research and to increase acceptance of the importance of metadata for basic science and industry. A number of articles, common regulations and community-driven suggestions regarding minimal requirements of metadata have been published during the last decade. Most of the well-known repositories used for microbiome data, such as MG-RAST, ENA and SRA/NCBI are all based on the MIxS (minimum information about any (X) sequence) checklist for reporting information about a nucleotide sequence (Yilmaz et al. 2011) (Additional file 1: Box 1). This checklist was developed by the Genomic Standards Consortium. The suggested standards specifically describe metagenomic and marker gene-based data sets, as part of the wider MIxS standard (Yilmaz et al. 2011). The respective website (Genomic Standards Consortium 2016) maintains up-to-date metadata checklists for genomes (MIGS), metagenomes (MIMS) and for marker genes (MIMARKS). The metadata suggested includes 11 items that are required for all kinds of submitted data: investigation type, project name, geographic location (latitude, longitude, country and/or sea, region), collection date, environment (biome, feature, material), environmental package and sequencing method. Similar DELSA Global (Data-Enabled Life Sciences Alliance) propose a simplified, yet informative and flexible multi-omics checklist in order to capture the essential aspects of omics studies (Kolker et al. 2014). It contains a number of questions regarding experiment information, design and methods, as well as data processing. Mainly in microbiome research, where the progress in available methodology has strongly influenced the field from the very beginning, it seems essential to implement a detailed description of methods and instruments used, to ensure the best possible reuse of data. When data is submitted to ENA/NCBI in addition to the MIxS requirements, a number of additional data must be added, depending on the environmental package selected. The minimal required data will consequently differ, depending on the research question. In addition, data generators are responsible that these data remain available, e.g., by making codes available.

Based on these observations, we believe that the minimum metadata requirements are essential for organizing metadata in microbiome research as opposed to the development of common metadata standards. Changing the current system is necessary, but it will be done gradually and will have to be constantly improved/re-thought. Due to rapid technological advances, current data has the potential to rapidly become obsolete, no matter how good the metadata is, preventing their re-use anyway. Moreover, metadata themselves will evolve depending on new parameters that we do not yet know are highly important in certain ecosystems. Nevertheless, there are bottlenecks of the existing requirements that need to be discussed. This includes ethical and legal issues as well as questions about how to make the submitting process of metadata and the recovery more user friendly.

## Ethical and legal issues of metadata standards

There is a common controversy among all areas of scientific research regarding data sharing. On the one hand data sharing is highly desirable as it increases the pace of knowledge discovery and scientific progress. On the other hand, it often poses challenges to the scientific community [14, 44]. Those challenges, which could be of ethical, cultural, legal, financial, or technical nature, must be considered when discussing the minimal metadata requirements for microbiome research. For example, dealing with clinical trials and patients’ data raises ethical and legal issues related to data de-identification and their possible re-identification [14]. This poses the question, whether human microbiome-related minimal metadata should/can include such sensitive information on the sample. Another example are the GPS coordinates of the sampling location. In most repositories, GPS coordinates are part of the essential metadata that must be submitted together with the uploaded sequence. While there is no doubt about the necessity of information about the sample location in most of the cases, there are situations in which they cannot be included. Examples include situations where accurate coordinates are not available due to governmental restrictions in certain countries/regions, the exact location is subject of intellectual property protection or of data protection issues not to interfere with the right of land owners, etc. These issues arise, for example, when private owners are unwilling to provide the exact location of their facilities in order to avoid negative associations with their business. This is especially relevant for datasets where high levels of pathogens or antibiotic resistance genes are found. Then again researchers from the industry sector might not accept if data on specific field sites is publicly available, for example if new plant cultivars are tested in trials in which new breeding efforts are made. Another important aspect, in the context of biological data and microbiome research, is the concern to publish collection coordinates for endangered species (e.g. those on the red lists). There is currently an ongoing debate on how to protect these species from poaching / illegal collection. Finally, also governmental organizations might not want exact locations of geopolitically important locations or contaminated sites, etc., to become public.

Other metadata information asked by the repositories may be a subject of intellectual property protection. For example, defined microbial consortia or the way how the sample was processed may be a non-disclosure information due to a patent protection [49]. Thus, we believe that the ideal minimum requirements for the metadata should allow for a maximum of the necessary information to be provided and, at the same time, tolerate alternative answers to the posed questions. Examples of such a solution may be permitting skipping the GPS coordinates question or exchanging it, for example, with the “country and region” field, or other relevant information.

The sharing of sequence data is currently seriously challenged by a considerable number of countries that claim sovereignty over all nucleotide sequences originating from genetic resources within their national borders and aim to gain control over the access to these sequences and that have therefore excluded them from public databases (Scholz et al. 2021). Unfortunately, worldwide many microbiome scientists are not aware of the Nagoya Protocol regulations (https://www.cbd.int/abs/) and the ongoing international negotiations of digital sequence information (https://www.cbd.int/dsi-gr/) negotiated in frame of the Convention on Biological Diversity (CBD). Due to the immense impact for microbiome sampling and data exploitation and (digital) storage this issue requires more awareness in the community. Moreover, it needs an active involvement and contribution of the scientific community to the discussion process, which is ongoing, and currently under debate. Due to diverging views of parties on this matter, a science and policy-based process was established recently; more information and documents can be found on the CBD website (https://www.cbd.int/dsi-gr/whatdone.shtml). The outcome of the debate will certainly influence the global metadata system.

## The need for a common digital identifier for metadata

A digital identifier is a unique name for the metadata record that allows finding metadata and connecting them to the respective dataset. In order for the metadata to be accessible even if the URL or the physical repository changes the address, a globally unique timeless identifier should exist for each metadata record [14]. Different organizations worldwide supply various globally unique persistent identifiers. For example, http://identifiers.org, provides resolvable identifiers in the form of URIs and CURIEs, while http://www.doi.org provides Digital Object Identifier (DOI). The DOI system run by the official DOI Registration Agency Crossref (www.crossref.org) is one of the most commonly used possibilities to identify the metadata and to connect it to the actual data. The DOI system is currently used mostly for crosslinking the metadata with the corresponding written materials such as journals, articles and books.

There is a large number of different metadata identifiers in microbiome research that are associated with diverse data repositories. This large variation in metadata identifiers makes taxonomic identification and comparisons between the studies rather complicated [53]. The most prominent example of digital identifiers in biology was established by the NCBI database that uses Sequence Read Archive (SRA) accession numbers for its repository of high throughput sequencing data. The NCBI SRA accession number is the most common identifier for sequencing studies as it is valid across the three most commonly used databases that are part of the International Nucleotide Sequence Database Collaboration (INSDC). INSDC includes NCBI SRA, European Bioinformatics Institute (EBI), and DNA Database of Japan (DDBJ). Other examples of the digital identifiers for omics data are the MG-RAST ID for data from DNA/RNA sequencing experiments processed by the MG-RAST pipeline, and the UniProt database for protein sequences.

Standardization of the use of digital identifiers among the omics-related data would greatly improve our ability to crosslink the metadata and to re-use available data. However, we endorse the establishment of one common globally valid digital identifier across all available sequencing databases. As the DOI system has established itself throughout the world as the most commonly used identifier for various kinds of information, some authors recommend using it also as a global persistent digital identifier for all sequencing studies [53].

## Common ontology

**Fig. 1:**
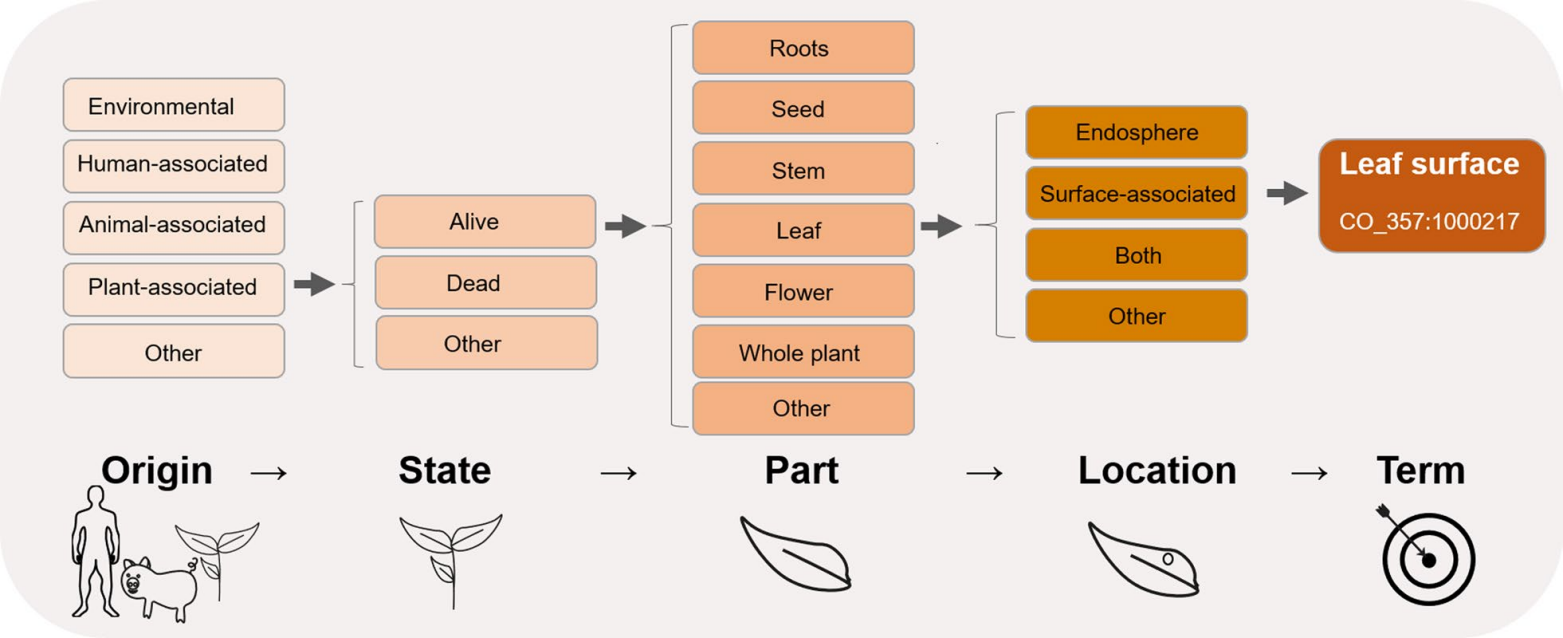
Example of the hierarchically built structure of the questions in the metadata application allowing for an easy and non-redundant ontology search for plant-associated microbiomes

Common ontology or technically correct words that are used to describe the host organism or environment are a key for metadata re-use. Ontologies are hierarchies of well-defined and standardized vocabulary interconnected by logical relationships (Bodenreider and Stevens 2006). Their main purpose is to make metadata searchable, comparable and machine-readable. In practice, however, researchers often complain about a non-stringent use of common ontologies. For example, in the case of plant root endophytes, the origin of the same sample may be described as “roots”, “root interior”, “root system” or more technically like “surface sterilized roots”. Also, information about the exact location might be added, like “root hair zone,” “lateral root zone”, etc. In the field of gut microbiome research, a sample dataset deposited as “mouse gut metagenome” could be of a gnotobiotic mouse colonized with a few bacterial species or with a microbial community of different origin (*e.g.*, soil, human feces), which is completely misleading. Finding the correct ontology for submitting metadata is therefore challenging as it needs to include all information but should at the same time allow for the retrieval of the data using simple search strings. As a consequence, many researchers submit only minimum required data instead of adding rich metadata, or use incorrect terms to describe their experimental data. One possibility for an easy and non-redundant ontology search is to structure the finding of a correct ontology in a hierarchical way. An example of such a hierarchical search for correct ontology is shown in Fig. 1. One solution is already available: synonyms in prokaryotic nomenclature are already linked in the LPSN-database (https://lpsn.dsmz.de). So, by connecting to this database, the issue of synonyms can be readily solved.

## From minimal data to FAIR principles

**Fig. 2:**
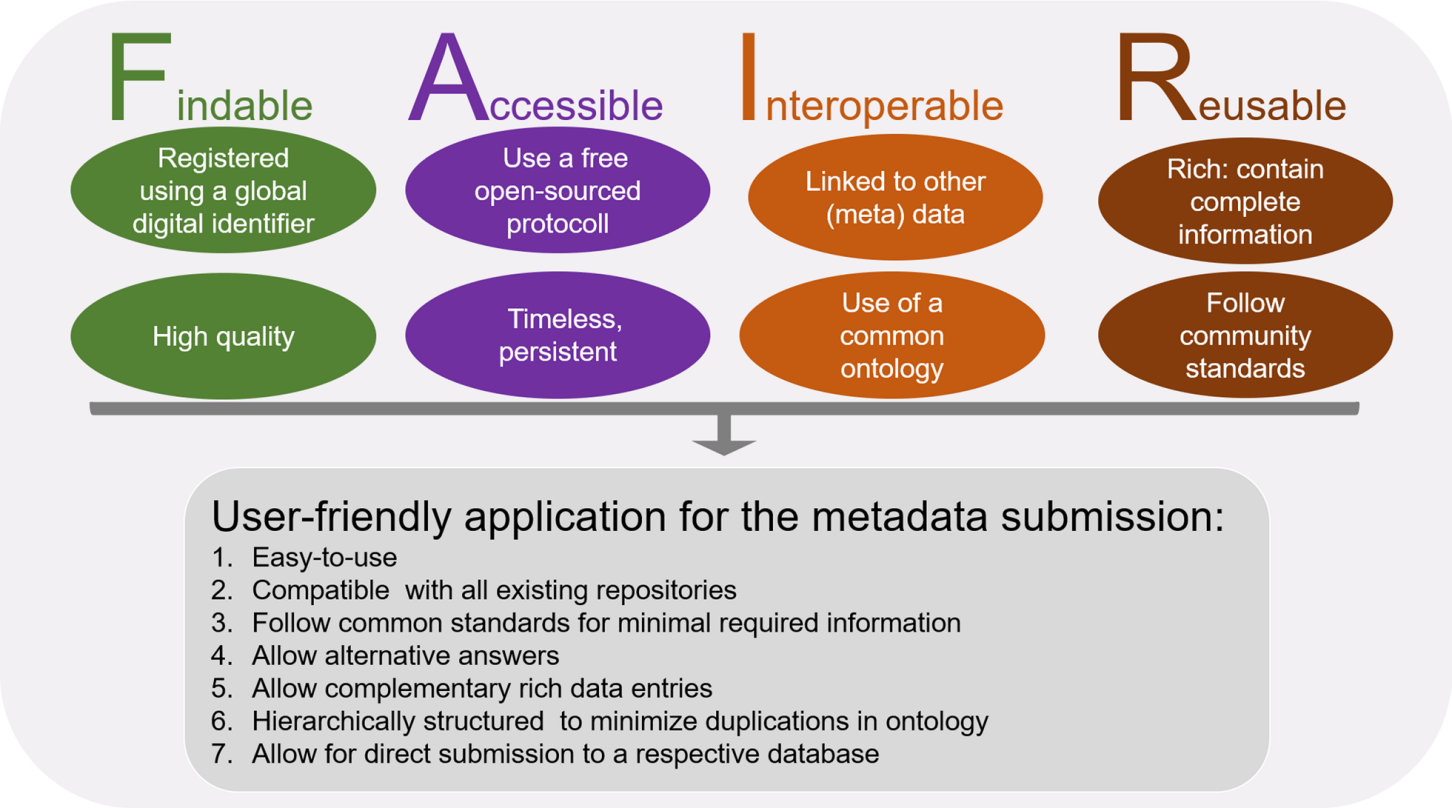
An overview of FAIR principles ( adapted from [56] that could be implemented in metadata requirements to facilitate search and re-use of deposited microbiome data in the future

Metadata for microbiome research data should contain essential information on the sample origin and processing as well as on the sequencing and bioinformatics methods, and used in such a manner that would allow researchers to compare data across various research fields and ensure their re-use. Other important parameters are that the metadata must be easily searchable, findable and searchable, freely available and contain information on the storage of the original sample. The use of a common ontology in the metadata descriptions must be simplified and standardized through providing an intuitive interface of the corresponding application. Crosslinking of the data and metadata should be done with one type of a timeless digital identifier, such as for example DOI, that should apply to all omics studies so that the data can be found and used in the distant future. The FAIR principles [56] (https://www.go-fair.org/fair-principles/), which are summarized in Fig. 2 would adequately address all these needs. Microbiome metadata following the FAIR principles will be: (1) easy to use and clearly structured in a hierarchical way; (2) compatible with all existing microbiome data repositories; (3) follow common standards for minimal required information; (4) allow replacement of some fields even belonging to the minimal required data with similar alternative questions (e.g. exact GPS coordinates of the sample location can be replaced with a region where the sample has been collected).

The questions implemented in the 'environmental packages' developed by the Genomic Standards Consortium (Yilmaz et al. 2011) could be used in the application to guide the researcher through the questions related to his/her study in a hierarchical manner, while the fields relevant for the specific research area have to be mandatory. The redundancy of the classification could be minimized by a hierarchical search option for a correct ontology integrated in the application. Finally, the output of the entry could be either a direct submission of the metadata in the selected repository or a correctly filled metadata spreadsheet that can be used for the same purpose.

Implementing the basic criteria of FAIR principles would allow an easy and uncomplicated entry of the complete metadata to each specific kind of a research question and at the same time ensure that datasets can be easily found by using simple search strings. At least, data-deposition is now a stringent criterion for publishing and use of FAIR principles should be enforced by publishers, editors and reviewers as well.

We believe that correctly filled metadata containing answers to minimal required questions should be obligatory for the submission of all sequence data and specifically verified before acceptance of the corresponding publication by all scientific journals in the future to improve data sharing between scientists.

## Selected use cases demonstrating added value of re-using microbiome datasets

Omics datasets are mainly produced to answer a specific scientific question. Only a small proportion of microbiome datasets were re-used after their initial publication or found in meta-studies and global assessments. Due to the fact that global standards for minimal required metadata in microbiome research are not yet available, re-use of available datasets can be a challenging and time-consuming task. It often requires that the dataset creators be contacted for missing information, which is especially a lengthy task when not only one but multiple datasets are re-used. Due to advancements in big data analyses and artificial intelligence, which are occurring in parallel to microbiome research [3, 11], it will become increasingly important to create added value by combining both. Integrating large numbers of available datasets to newly developed algorithms based on machine learning with improved analytical capacity can help to improve our understanding and therapeutic possibilities of poorly understood diseases that might be linked to the microbiota, for example inflammatory bowel disease in humans [9]. In a recent study, Pinart et al. (2021) compared observational studies with data on nutrition and gut microbiome composition based on a template from the European Nutritional Phenotype Assessment and Data Sharing Initiative (ENPADASI) consortium. Only a few studies were found which contain information on both dietary intake and the gut microbiome. Thus, development of more readily retrievable information from metadata will be essential for combined applications in the future. Although data generated in microbiome research is currently sparsely re-used, the benefit of such attempts is illustrated by selected use cases that led to important discoveries. In plant microbiome research, microbial communities inside seeds came recently into the spotlight due to their implications for host health and fitness, as well as their potential for biotechnological applications (Berg & Raaijmakers, 2019). Simonin et al. [50] have conducted the first meta-analysis of seed microbiome datasets by retrieving data from 63 studies that encompassed 50 different plant species. They re-analyzed raw data from a number of metabarcoding studies, which were obtained from public databases and used 16S rRNA gene fragments, *gyrB*, and the fungal ITS region s genetic markers for microbial community profiling. By conducting this study, the authors could identify plant species that harbor highly diverse microbial communities inside their seeds. Moreover, by applying this large-scale approach, they also identified seed microorganisms that are shared by a wide range of phylogenetically unrelated plant species. Such microorganisms likely have an intrinsic, evolutionary conserved association with plant hosts that would have remained undiscovered without this study. The ongoing exploitation of this meta-analysis resulted in the identification of bacterial and fungal core taxa, such as *Pantoea agglomerans*, *Pseudomonas viridiflava, P. fluorescens*, *Cladosporium perangustum* and *Alternaria sp.,* [50]. Another study, based on short-read metagenomics, addressed environmental resistomes in bog ecosystems. Obermeier and colleagues [30] found that such untouched ecosystems harbor highly diverse resistomes. In order to verify this so-far undescribed and surprising observation, the authors had to compare their data with other publicly available datasets from the same ecosystem but geographically distant sites. Their data were compared with previously established culture collections from Germany and Norway [32, 31] as well as with Austrian and Swedish metagenomes [8, 7, 27, 29, 57]. This comparative study confirmed the initial hypothesis of naturally occurring antimicrobial resistances in pristine environments and facilitated the development of a new theory about resistome development in natural habitats. Metagenomics data sets mentioned above were further exploited, and yielded in basic discoveries for plant microbiome science, e.g. deciphering role of *Archaea* on plants (Taffner et al. 2018), and understanding different plant-microbiome coevolution for mosses and vascular plants [55].

Metagenome-assembled genomes (MAGs) provide the possibility to analyze short read-based datasets at species-level or even strain-level by applying certain assembly and binning strategies. In a recent study, 303 metagenomes obtained from fermented foods were used for MAG reconstruction and compared to 9445 publicly available human metagenomes that were subjected to the same processing strategy [34]. The authors discovered that a certain proportion of lactic acid bacteria in the human gut is likely obtained from fermented foods, while other bacteria from this group are acquired via different routes. In another study that was based on comparative MAG analyses, the authors have reconstructed 498 MAGs from paleofaeces samples and compared them to publicly available metagenomes obtained from recent stool samples from different geographic regions [54]. They not only found that the ancient samples (1000–2000 years old) were more similar to present-day non-industrialized human gut samples, but they also located important symbionts that have undergone changes during adaptation processes over the past centuries.

The presented use cases are not exhaustive, but they clearly show that re-use of multiple datasets produced in different microbiome studies can be of added value by providing insights that would not be possible by solely relying on datasets that were obtained in one specific study. Their increased implementation will become more feasible if global standards for minimal required metadata based on FAIR principles are developed and broadly applied in the future. In future, re-using microbiome datasets will contribute to promoting complex and mechanistic microbiome studies.

## Conclusions

The science community needs globally accepted common standards, especially in fast-developing areas such as microbiome research to ensure best possible use, re-use and integration of data. To make the process of metadata submission uncomplicated, database-independent and thus allow for a higher quality of submitted metadata, we propose the development of a globally recognized metadata submission tool. The proposed application should fulfill important FAIR Guiding Principles, be user-friendly, compatible with all existing repositories, follow common standards for minimal required information, allow alternative answers, allow complementary rich data entries, be hierarchically structured to avoid duplications in ontology and allow for direct submission to a respective database. Developing of global standards for the minimal required metadata in microbiome research should involve international collaboration between research institutions, bioinformaticians and data repositories all over the world. It would also require dedicated funding, manpower and infrastructure for this purpose, which some countries have already begun to provide. The most important hurdle are legal issues arising from the Convention on Biological Diversity (CBD), especially from the Nagoya Protocol on Access and Benefit-sharing; here more attention and involvement of the scientific community is required. Despite of all challenges, we believe that a common and competitive solution for the standardization of metadata in microbiome research is necessary for retrieving and using of existing and novel sequencing data in this fast-developing research field.

## Supplementary Information

**Additional file 1.** Supplemental information.

## Data Availability

Not applicable.
