# Supplementary material for: Metadata harmonization–Standards are the key for a better usage of omics data for integrative microbiome analysis
Source: Environ Microbiome. 2022 Jun 24;17:33. doi: 10.1186/s40793-022-00425-1 (PMC9233336; doi:10.1186/s40793-022-00425-1)
Supplement: Supplementary file 1 — Additional file 1. Supplemental information. [file 40793_2022_425_MOESM1_ESM.docx]

**Box 1.** Minimal metadata requirements for selected microbiome data repositories and standards

1. Minimal required information about the study and geographical location

|  | **Project Information** | | | | **Extra** |
| --- | --- | --- | --- | --- | --- |
|  | **Project name/title** | **Investigation type/project data type** | **Study summary/description** | **Authors/ organization** |  |
| **Example** | test name | Metagenome | study summary | Name(s), organization information |  |
| **ENA/NCBI ^1^** | + | (+) | + | + |  |
| **MG-RAST ^2^** | + | + | - | + |  |
| **MIxS ^3^** | + | + | - | - |  |
| **Biodiversity Exploratories ^4^** | + | + | + | + | Plot number; data structure; variable descriptions |

1. Minimal required information related to the geographical location and collection date

|  | **Geographical location** | | |  | **Collection date** | **Collection time/time zone** |
| --- | --- | --- | --- | --- | --- | --- |
|  | **latitude and longitude** | **altitude/elevation** | **Country and/ or sea** | **location** |  |  |
| **Example** | GPS coordinates | Depth or elevation | Country | Specific local name | date |  |
| **ENA/NCBI** | (+) | - | (+) | - | + | - |
| **MG-RAST** | + | - | + | + | + | + |
| **MIxS** | + |  |  |  | + | - |
| **Biodiversity Exploratories** | + | + | - |  | + | + |

1. Minimal required information about the sample: Environmental information

|  | **Biome/ env_broad_scale/ habitat** | **Env. Feature/ env_local_scale/ Environmental descriptors** | **Env. Material/ env_medium/ Biotic data type** | **Env. Package** |
| --- | --- | --- | --- | --- |
| **Example** | Rhizosphere | Agricultural field | soil | plant-associated |
| **ENA/NCBI** | (+) | (+) | (+) | (+) |
| **MG-RAST** | + | + | + | + |
| **MIxS** | + | + | + | (-) |
| **Biodiversity Exploratories** | + | + |  |  |

1. Minimal required information about the sample: Sampling information

|  | **Organism/ environment/ taxa** | **Number of plots** | **Experimental manipulation** | **Measurements/ Repeated measurement** | **Processes and services** | **Equipment** |
| --- | --- | --- | --- | --- | --- | --- |
| **ENA/NCBI** | (+) | - | - | - | - | - |
| **MG-RAST** | - | - | - | - | - | - |
| **MIxS** | - | - | - | - | - | - |
| **Biodiversity Exploratories** | + | + | + | + | + | + |

1. Minimal required sequencing information

|  | **Metagenome name** | **Sequencing method/platform** | **Extra** |
| --- | --- | --- | --- |
| **Example** | Test ID | ILLUMINA |  |
| **ENA/NCBI*** | - | (+) | Library strategy (amplicon), library source (metagenomic), library selection (PCR), library layout (paired), platform (Illumina), instrument model (fastq), design description ^5^ |
| **MG-RAST** | + | + |  |
| **MIxS** | - | + |  |
| **Biodiversity Exploratories** | - | - |  |

^1^ The minimal required information for the ENA/NCBI repository was obtained from the <https://www.ebi.ac.uk/ena/browser/checklists> website. As the minimum required information at the ENA/NCBI repository varies strongly depending on the sample type, the (+) sign was used to describe the data fields that were required by the majority of the host/environmental-associated microbiome-related data. The specific sample types, such as pathogens-related information, or non-microbial samples were excluded from the Table.

^2^ MG-RAST minimum required information was obtained from the template available at <https://www.mg-rast.org/mgmain.html?mgpage=upload>

^3^ The information about the minimal required metadata MIxS was obtained from <https://gensc.org/mixs/>, while only the fields mandatory for all kinds of data were included.

^4^ The information about metadata minimum required information for the Biodiversity Exploratories was obtained from <https://www.bexis.uni-jena.de>

^5^ The minimal required data by the SRA metadata spreadsheet is available at <https://www.ncbi.nlm.nih.gov/sra/docs/submitmeta/>
